# An integrative review protocol on interventions to improve users’ ability to identify trustworthy online health information

**DOI:** 10.1371/journal.pone.0284028

**Published:** 2023-04-06

**Authors:** Hind Mohamed, Jon Salsberg, Dervla Kelly

**Affiliations:** 1 School of Medicine, University of Limerick, Limerick, Ireland; 2 ULCaN and HIST Research Clusters, Health Research Institute, University of Limerick, Limerick, Ireland; Federal University of Piaui: Universidade Federal do Piaui, BRAZIL

## Abstract

**Background:**

The epidemiological transition phenomena drive the attention to focus the scope on health literacy as it has an impact on patients’ health outcomes and quality of life.

**Aim:**

This paper aims to explore the implemented interventions for improving users’ ability to identify trustworthy online health information.

**Methodology:**

A comprehensive search of the literature will be conducted on the following electronic bibliographic databases: Ovid Medline, Embase, Cochrane database, Academic search complete and APA psycinfo. Further, manual search of eligible studies reference lists will be carried out to identify other eligible studies. The search strategy will include a combination of three key blocks of terms, namely: (adult OR adults) Or (patient OR patients) OR (layperson OR laypersons) OR (caregiver OR caregivers), (Intervention OR Interventions) OR Educational programs OR (health literacy And curriculum) OR Community outreach OR Interactive workshops OR (Online portal OR Patient Portals), and information seeking behavior OR consumer health information OR online information OR social media OR access to information. The results of these categories will then be combined using the AND connector. Two independent reviewers will screen and assess data quality. Disagreements will be resolved by consensus. Due to the anticipated methodological pluralism of the potentially eligible studies, a narrative synthesis of the findings on interventions aimed at improving users’ ability to identify trustworthy online information will be provided according to the pre-identified thematic areas. Furthermore, a narrative synthesis of the reported barriers and facilitators for applying these interventions by end users.

**Expected results and impact:**

Given that the focus of our review findings is on understanding the breadth and depth of the global research into interventions to improve users’ ability to identify trustworthy online health information. The findings will be of great value to inform future innovative approaches to promote identification of trustable online sources for young people worldwide.

## Introduction

The World Health Organization defines health literacy as "the cognitive and social skills which determine the motivation and ability of individuals to gain access to, understand and use information in ways which promote and maintain good health." Unfortunately, people with limited health literacy tend to have lower efficacy for maintaining healthy behaviours, leading to increased incidence of chronic diseases and health care costs. Nowadays, more and more information are distributed digitally, and the term digital health literacy can be used to describe health information in this context [1].

The value of digital health lies in the use of information and communication technologies for improving patients’ health and well-being and the delivery of health care services. Poor accessibility of health information in digital formats can lead to multiple diagnostic, therapeutic, and prognostic harms, which have an impact on patient safety [2].

Misinformation is defined as information, which is distributed with no intention to cause harm, in contrast to disinformation, where there is an intention to deceive. The spread of misinformation occurs through social contacts or social media [3]. Everyone has now become a potential dissemination source with the capacity to share whatever they believe regardless of its reliability [4]. Moreover, the interconnectivity of the digital era means that the rapid spread of misinformation has a corrosive power on the individual’s short-, medium- and long-term health outcomes [3].

Previous studies identified many factors that play essential roles in determining an individual’s online information-seeking behaviour, such as demographic characteristics (age, gender, educational, socioeconomic, and employment status) and health status [5]. Elderly patients are reluctant to use the Internet, which is consistent with offline gender-based health-seeking behaviour [6]. Individuals who are better educated, employed, and of a higher social class prefer to use online websites as a source of health information [7]. Those with poor or fair health, chronic conditions, and a long-term illness or disability were more likely to visit health sites than those who reported better health [8]. A study on rural digital immigrants in China [9] reported that poor information skills, lack of health information literacy, and low readability of online health information are significant barriers for digital immigrants to seeking health information via the Internet.

Although the Internet offers many advantages to consumers of online health information, searching for specific information can cause many issues concerning the credibility of the information provided [10]. Finding high-quality sites can be challenging, and there are problems with author identification, completeness, and accuracy of the information provided [11,12].

Evaluating the reliability of online health information has been a central issue in consumer health informatics for many years [13]. However, a recent review done in Finland (2020) [14] showed that many adolescents face a challenge in evaluating the credibility of health-related online information. Although they understand that online information is not always to be trusted, many remain unsure of how to evaluate its credibility [15–17].

A 2020 systematic review study [18] identified 17 tools for assessing the trustworthiness of online information, all published between 1997 and 2018 (S1 Table). The tools were developed for different purposes, from a general quality assessment of medical information to detailed analyses. However, the development process of the tools was poorly described. Some tools lacked essential criteria for assessing the trustworthiness of medical information and were not designed for a lay audience.

The criteria used for evaluating online website information in the literature vary depending on the population and context but often include website position in search results, picture quality; celebrity endorsement; website readability; website authorship; website credentials, and consistency with search intentions [19]. A study done in the United States [11] identified twenty-five evaluation criteria (S2 Table). However, using the evaluation criteria for online health information depends on consumers’ health literacy and educational level [19]. For example, Mackert et al. showed that individuals with low health literacy used "position in search results, quality of pictures, celebrity endorsement, and website authorship as criteria to evaluate online health information [13].

A 2019 systematic review [11] done in the United States identified the common indicators used by internet consumers to evaluate the quality of online health information, which are related to three aspects of online information: source, content, and design. Studies done by Fogg et al. (2003) [20] and Walthen and Burkell (2002) [21] reported that most consumers of online information depend on simple methods for credibility evaluation, relying on site presentation and visual design rather than considering content and source presentation.

Accordingly, many information processing theories have been used to explain information-seeking behaviours for users’ credibility assessment of web-based information [22]. For example, the dual processing model stated that internet consumers use either central or peripheral strategies for information evaluation depending on high or low motivational status, respectively [23]. This was supported by the limited capacity model of message processing, which stated that people usually process part of the message provided as they have a limited cognitive capacity for message processing [24]. Additionally, the information-foraging theory claims that whenever feasible, humans will choose behaviours that ’’tend to optimize the utility of information gained as a function of interaction cost’’ [25,26]. Conclusively and taken together, online information seekers tend to use strategies that minimise their cognitive and time investments to cope with the high load of available information.

Many interventions exist targeting a lay person’s ability to find reliable online information. For example, a 2014 study [27] identified the importance of educational programs, interactive workshops, collaborative learning, health literacy curriculum, community outreach, and an online portal with support via videoconferencing. All these interventions aim to teach people how to navigate online, then measure changes in knowledge and internet skills for evaluation purposes.

There is a need to think about a new form of social behaviour surrounding information-seeking that accounts for economic and social effects and requires a change in how science and information professionals approach the dissemination of knowledge. Above that, actions to combat misinformation must be planned and carried out with the support of patients and public.

### Aim and objectives

#### Aim

Explore the implemented interventions to improve users’ ability to identify trustworthy online health information.

#### Objectives

1. Understand the types of implemented interventions for improving users’ ability to identify trustworthy digital health information.
2. Understand the outcomes of the implemented interventions for improving user’s attitude and behaviour to identify trustworthy online health information.
3. Understand the outcomes of the implemented interventions for improving user’s knowledge about their health.
4. Explore the measures by which these interventions are evaluated.
5. Understand the barriers and facilitators for using these interventions by consumers
6. Explore the effectiveness of these interventions for helping users to identify trustworthy online health information.

## Materials and methods

Ethics approval and consent to participate is not required for conduction of this integrative systematic review. As this study is solely based on the analysis of previously published anonymous data and contain no identifiable individual person’s data, therefore consent to participate is not applicable.

This systematic review will be reported based on the PRISMA (Preferred Reporting Items for Systematic Reviews and Meta-Analysis) S1 Checklist [28].

### Review questions

PICO (population, intervention, comparator, outcome) framework was used to build our research question [29] as follows:

**Population:** Adult patients and their carer’s, and lay persons (19 to 65 years).

**Intervention:** Implemented interventions to improve users’ ability to identify trustworthy online health information. Examples of potential inclusions are educational programs, community outreach, health literacy curriculum, and university-based health programs.

**Comparator:** Studies with or without comparative groups will be considered for the review.

**Primary outcome:** Understand the outcomes of the implemented interventions for improving user’s attitude and behaviour to identify trustworthy online health information.

**Secondary outcomes**: Understand the outcomes of the implemented interventions for improving user’s knowledge about their health.

The systematic integrative review will address the following questions:

1. What interventions have been implemented to improve users’ ability to identify trustworthy online health information?
2. What are the outcomes of the implemented interventions for improving users’ ability to identify trustworthy online health information?
3. What are the outcomes of the implemented interventions for improving users’ knowledge about their health?
4. What are the reported barriers and facilitators for using those interventions by adults?
5. How effective are those interventions for helping users to identify trustworthy online health information?

### Study design

This integrative review will include peer-reviewed quantitative (experimental and observational), qualitative, and mixed-methods studies.

### Context

Any study reporting an intervention conducted among adult people globally will be considered for the review (Community-based, Institutional, any specific setting based).

### Eligibility criteria

Table 1 show the inclusion and exclusion criteria with the justification.

**Table 1:** Inclusion and exclusion criteria with justification.

Criterion	Inclusion	Exclusion	Justification
Sample	Human studies	Animal studies	Referring to patient and public involvement in health research.
Population	• Adult group of patients, carers, and the public. • Studies targeting adult age group (19 to 65 years)	Healthcare providers	Explore implemented interventions targeting lay people.
Language	English, Spanish and Portuguese	Other languages	To widening the scope of the findings.
Time period	Studies done between 2006–2023	Studies done outside this time frame	Covering a wide range of literature for implemented interventions during this time frame (more than 90%)
Geographic location	International context		Identify locations where interventions were implemented targeting lay people.
Study focus	• Articles that discuss implemented interventions for improving laypersons’ ability to identify trustworthy online health information • Articles that present information on health literacy before and after the intervention.	• Studies focusing on unimplemented programmes such as polices, laws and regulations of governmental and non-governmental organisations • Articles targeting interventions for healthcare providers. • Articles that don’t present information on health literacy before and after the intervention.	• Referring to the context of the interventions. Explore interventions targeting online information seeking behaviour for lay people in any setting (Offline, Online). • Identify gaps for using the implemented interventions by lay people to help improving the design of future interventions.
Type of article	Peer reviewed journal articles (Qualitative, quantitative studies, mixed methods) and Randomised and non-randomised controlled trials	Grey literature and website’s publications	Time constraints and feasibility

### Information sources

We will explore several electronic databases, including Ovid Medline, Embase, Cochrane database, Academic search complete and APA psycinfo. In addition, a discussion with two additional research team members regarding exclusion and inclusion criteria at the outset of the systematic integrative review process will occur. Finally, we will manually search the references of the included studies and track their citations to identify other eligible studies.

### Search strategy

A health sciences librarian helped to construct the search strategy. Medical Subject Headings (MESH) and free-text terms will be used. Truncation and adjacency searching are used to increase the sensitivity of the search, as appropriate.

A preliminary initial search was conducted on the Medline database to identify the mesh terms related to our research questions as follows:

(adult OR adults) OR (patient OR patients) OR (layperson OR laypersons) OR (caregiver OR caregivers).

(Intervention OR interventions) OR Educational programs OR (health literacy And

curriculum) OR Community outreach OR Interactive workshops OR (Online portal OR Patient Portals).

Information seeking behaviour OR consumer health information OR online information OR social media OR Access to information.

The results of these categories were then combined using the AND connector.

## Data management

Duplicates will be removed using Endnote Reference Management Software (Clarivate), and additional duplicates not identified by the Endnote function can be removed manually. The deduplicated data can then be imported into Covidence (Veritas Health Innovation Ltd), a review-management software program that operates in partnership with Cochrane Collaboration and allows multiple reviewers to work on study selection simultaneously and independently.

### Study selection

The eligible studies will be identified in 2 stages: title and abstract screening and full-text review. Abstracts and titles will be screened by two independent reviewers using the eligibility criteria outlined above. Conflicts will be resolved by discussion and adjudicated by a third independent reviewer when necessary. The full-text review of all studies selected during the screening will be independently conducted by two reviewers, with disagreement resolved as described previously.

### Data extraction

Two reviewers will independently extract the data from each eligible study using a Microsoft Excel data extraction form. Other reviewers can review the extracted data to ensure accuracy and completeness of the data. The following study details will be extracted: authors, year of publication, country, affiliation, study aim, study design, and publication status. Data will also be extracted about types of implemented interventions to improve users’ ability to identify trustworthy digital health information, how they measure their success and effectiveness, and the barriers, facilitators, and outcomes of these interventions.

### Quality assessment

The mixed methods appraisal tool will be adopted to integrate evidence from quantitative, qualitative, and mixed-methods studies [30]. Two reviewers will appraise the quality of each study independently. Disagreements between reviewers can be resolved by discussion. The results of the quality appraisal will be presented for each study, and any eligible study will not be excluded based on the quality appraisal in keeping with the integrative review methodology [31].

### Data synthesis

Due to different methodological designs of potentially included reviews, the findings of the narrative synthesis will be provided from the included studies, structured around answering the main review questions. We will present the summarised information on interventions aimed at improving users’ ability to identify trustworthy online health information according to identified thematic areas such as the study setting, target population, type of intervention, intervention objectives, and how the interventions work. Furthermore, Whittemore and Knafl’s integrative review approach will be used as guidance for narrative synthesis [31].

Multiple different outcomes will be measured across various study designs; thus, a meta-analysis will not be feasible. The intervention’s outcomes will be summarised according to the reported change in the attitude and behaviour to identify trustworthy digital health information and health knowledge before and after the interventions. Furthermore, a narrative synthesis of the reported barriers and facilitators of the implemented interventions will be provided.

## Results and discussion

Patients or the public will be involved in synthesising and interpreting the study results. The findings of this research will be helpful for lay people to identify the gaps of implemented interventions and address the issues related to the barriers. Policymakers can also use the research findings to promote scaling up of the implemented interventions globally and facilitate designing of future approaches based on technology and innovations.

The results of the study will be disseminated through peer-reviewed publications, academic conferences, and to all patients and the public involved in this study. The findings will be disseminated to the public through local media (Radio) and newspapers articles.

## Conclusions

The findings of this paper will inform the design of future user-centred interventions tailored to patients and public needs.

## Supporting information

S1 Checklist PRISMA-P (Preferred Reporting Items for Systematic review and Meta-Analysis Protocols) 2015 checklist: Recommended items to address in a systematic review protocol*. (DOC)

S1 Table Tools for assessing trustworthiness of online health information. (DOCX)

S2 Table Criteria used for evaluation of online website information. (DOCX)
