# Supplementary material for: An integrative review protocol on interventions to improve users’ ability to identify trustworthy online health information
Source: PLoS One. 2023 Apr 6;18(4):e0284028. doi: 10.1371/journal.pone.0284028 (PMC10079012; doi:10.1371/journal.pone.0284028)
Supplement: S1 Table — (DOCX) [file pone.0284028.s002.docx]

**Supporting information**

**S1 Table. Tools for assessing trustworthiness of online health information**

| **No.** | **Tools [18]** |
| --- | --- |
| 1. | Silberg |
| 2. | Kapoun |
| 3. | Gillois |
| 4. | Jiang criteria |
| 5. | CART (Completeness, Accuracy, Relevance, Timeliness) |
| 6. | Sandvik scale |
| 7. | QUEST (Quality Evaluation Scoring Tool) |
| 8 | 11-Point Quality Assessment Scale |
| 9 | Aslani criteria |
| 10 | AMA (American Medical Association), |
| 11 | Grid ULiège |
| 12 | Trumble Tool |
| 13 | Banzi tool |
| 14 | OncoRX-IQ |
| 15 | HONcode |
| 16 | Joubert |
| 17 | eHealth Code of Ethics |
