# Supplementary material for: An integrative review protocol on interventions to improve users’ ability to identify trustworthy online health information
Source: PLoS One. 2023 Apr 6;18(4):e0284028. doi: 10.1371/journal.pone.0284028 (PMC10079012; doi:10.1371/journal.pone.0284028)
Supplement: S2 Table — (DOCX) [file pone.0284028.s003.docx]

**Supporting information**

**S2 Table. Criteria used for evaluation of online website information**

| **No.** | **Criterion [11]** |
| --- | --- |
| 1 | Trustworthiness |
| 2 | Expertise |
| 3 | Objectivity |
| 4 | Transparency |
| 5 | Popularity |
| 6 | Understandability |
| 7 | Relevance |
| 8 | Familiarity |
| 9 | Accessibility |
| 10 | Identification |
| 11 | Believability |
| 12 | Accuracy |
| 13 | Readability |
| 14 | Currency |
| 15 | Navigability |
| 16 | Aesthetics |
| 17 | Interactivity |
| 18 | Comprehensiveness |
| 19 | Practicality |
| 20 | Completeness |
| 21 | Usefulness |
| 22 | Balanced |
| 23 | Anonymity |
| 24 | Security |
| 25 | Learnability |
